# Discriminatory Ability of Hypervariable Variable Number Tandem Repeat Loci in Population-based Analysis of *Mycobacterium tuberculosis* Strains, London, UK

**DOI:** 10.3201/eid1510.090463

**Published:** 2009-10

**Authors:** Preya Velji, Vladyslav Nikolayevskyy, Timothy Brown, Francis Drobniewski

**Affiliations:** Barts and The London School of Medicine, Queen Mary, University of London, London, UK (P. Velji, V. Nikolayevskyy); Health Protection Agency National Mycobacterium Reference Laboratory, London (T. Brown, F. Drobniewski); 1 These authors contributed equally to this article.

**Keywords:** Tuberculosis, tuberculosis and other mycobacteria, metropolitan area, bacterial genetics, genotyping, reproducibility, bacteria, London, research

## Abstract

To address conflicting results about the stability of variable number tandem repeat (VNTR) loci and their value in prospective molecular epidemiology of *Mycobacterium tuberculosis*, we conducted a large prospective population-based analysis of all *M. tuberculosis* strains in a metropolitan setting. Optimal and reproducible conditions for reliable PCR and fragment analysis, comprising enzymes, denaturing conditions, and capillary temperature, were identified for a panel of hypervariable loci, including 3232, 2163a, 1982, and 4052. A total of 2,261 individual *M. tuberculosis* isolates and 265 sets of serial isolates were analyzed by using a standardized 15-loci VNTR panel, then an optimized hypervariable loci panel. The discriminative ability of loci varied substantially; locus VNTR 3232 varied the most, with 19 allelic variants and Hunter-Gaston index value of 0.909 unNN. Hypervariable loci should be included in standardized panels because they can provide consistent comparable results at multiple settings, provided the proposed conditions are adhered to.

## Abstract 2

Hypervariable loci should be included in standardized panels because they can provide consistent comparable results in multiple settings.

Globally, tuberculosis (TB) accounts for almost 2 million deaths each year (*1*). Although TB notification rates in the United Kingdom (13.8/100,000 in 2007) remain low, rates differ substantially by region: London (43.2/100,000) accounts for ≈40% of all TB cases registered in the United Kingdom, and ≈75% of TB patients in London were born abroad (*2*). Rates of drug resistance also are higher in London than in the rest of the United Kingdom: 8.6% of isolates are isoniazid resistant, and 1.2% are multidrug resistant (UK Health Protection Agency; www.hpa.org.uk).

In settings where incidence of TB is low or moderate, molecular genotyping is used to investigate suspected TB outbreaks, laboratory cross-contamination, and reactivation and (at a population level) to identify clustered cases that are not apparently linked; for the latter purpose, the highest possible level of discrimination is required (*3*). For these purposes, insertion sequence (IS) *6110* restriction fragment length polymorphism (RFLP) analysis—often supplemented with spoligotyping and, more recently, with variable number tandem repeat (VNTR) typing—is used routinely.

The highest levels of epidemiologic discrimination of strains of the *Mycobacterium tuberculosis* complex (MTBC) can be achieved by using multilocus VNTR typing, but these results depend on the number and loci used, particularly for homogenous strain groups such as the Beijing family (*3*–*5*). This approach overcomes technical difficulties associated with IS*6110*-RFLP and is amenable to automation that results in a high throughput (*6*–*10*). A standardized panel of 15 + 9 VNTR loci (24 loci) has been proposed (*7*,*11*), but it is unclear whether sufficient discrimination would be seen when the panel is used in populations with a substantial prevalence of homogenous MTBC families (*4*,*5*,*12*). In addition, the discriminative power of VNTR loci may vary markedly among genetic families (*7*,*13*). Recent studies evaluating the discriminative power of VNTR typing have produced conflicting results that were generated by using convenience samples (small populations with low diversity or populations confined to a single geographic setting). These studies highlighted a need for larger population-based studies to identify discriminative VNTR loci and ascertain their applicability for various genetic groups.

Concerns about the stability and reproducibility of particularly useful hypervariable loci, such as 3232, 2163a, 3336, and 1982 (*3*–*5*,*14*), have been raised (*7*,*15*). As a result, they have been excluded from the proposed international panels for VNTR typing. For these reasons, we conducted a study to examine the stability of hypervariable loci and the parameters associated with reproducibility, to select loci suitable for prospective molecular epidemiologic studies, and to evaluate the discriminatory power of these loci at a population level in a metropolitan setting.

## Materials and Methods

### Bacterial Isolates

A total of 2,261 individual MTBC isolates (1 per patient) were included in this prospectively designed population study. These isolates represented 95.7% of the bacteriologically confirmed TB cases reported from the 30 London hospitals in the 12 months from April 2005 through March 2006. These isolates had been characterized by using spoligotyping, and all but 4 were assigned to 1 of 36 spoligotype families (*16*,*17*). Multiple isolates were available from 265 patients (11.7%), resulting in serial isolate sets of 2–6 isolates, which had been sampled at intervals of 3 days to 11 months (N = 632).

### Multilocus VNTR Analysis

All extracts were typed by using 15 mycobacterial interspersed repetitive unit (MIRU)-VNTR loci as previously described (*3*). Isolates clustered when the 15 MIRU- Hunter-Gaston index value VNTR profiles we used were reanalyzed with an additional panel of VNTR loci 2163b, 2347, 3232, 2163a, 1982, 3336, and 4052 as previously described (*3*,*5*) after optimization of factors affecting reproducibility (see Hypervariable Loci Optimization). Variability or discrimination at a locus was assessed by using the Hunter-Gaston Discriminative Index (HGDI) (*18*). Loci with HGDI values <0.3, 0.3–0.6, and >0.6 were considered poorly, moderately, and highly discriminative, respectively (*19*).

### Hypervariable Loci Optimization

We selected 16 previously characterized MTBC isolates to cover the complete range of repeat sizes at control loci MIRU 26 and exact tandem repeat (ETR)–B and experimental hypervariable loci VNTRs 1982 and 3232 (except 0 repeats for the locus 3232). For each of the 16 extracts, four 10-µL PCRs were conducted for each of the primer mixes in duplicate. Of these 4 reactions, the first was performed as described previously with BIOTAQ polymerase (Bioline, London, UK) (any enzyme in the given context means enzyme in conjunction with the buffer recommended and supplied by a manufacturer). Three other sets of PCRs were conducted under different amplification conditions (*1*).

#### Method 1

Diamond DNA polymerase (Bioline) was used (*9*). The PCR amplification cycle was 3 min at 95°C, followed by 35 cycles of 30 s at 95°C, 30 s at 60°C, and 2 min at 72°C, and 1 final cycle of 5 min at 72°C (*2*).

#### Method 2

HotStartTaq DNA polymerase (QIAGEN, Hilden Germany) was used. Each 10-µL reaction contained 1× PCR buffer (QIAGEN), 0.25 U/µL of the relevant polymerase, 0.2 µmol/L dNTPs, 0.125 µmol/L of relevant primer, and 5% dimethylsulfoxide. The DNA amplification cycle was 15 min at 95°C, followed by 35 cycles of 30 s at 94°C, 30 s at 60°C, and 1 min at 72°C, and a final cycle of 10 min at 72°C (*3*).

#### Method 3

HotStartTaq Plus DNA polymerase (QIAGEN) was used. The PCR mixture was the same as in method 2, and the amplification cycle was the same, except that the initial 95°C activation time was reduced to 5 min.

We manually calculated the number of repeats within each PCR product by resolving 4 µLof each product on a 1.2% (wt/vol) agarose gel (Agarose LE Analytical grade; Promega, Southampton, UK) against a 2,000-bp HyperLadder II standard (Bioline). The number of repeats at each locus also was calculated by sizing in a denaturing capillary electrophoresis system using a CEQ 8000 instrument with a DNA Size Standard 600 (Beckman Coulter, High Wycombe, UK) and MapMarker D1 labeled 640–1000 (BioVentures, Inc., Murfreesboro, TN, USA) because fragments were expected to be >600 bp. Three parameter sets (Table 1) were used to analyze all fragments. The different parameters examined were capillary temperature (60°C for methods 1 and 2 and 50°C for method 3, respectively), denaturation time (120 s for method 1 and 180 s for methods 2 and 3, respectively) and separation time (60 min for methods 1 and 2 and 70 min for method 3, respectively). Fragment data traces were automatically analyzed by using the scheme shown in Table 1. For locus 3232, we accounted for offset values (i.e., difference among actual sizes of PCR fragments and apparent sizes indicated by electrophoresis) when calculating number of repeats in Table 1.

**Table 1:** Expected molecular weights of *Mycobacterium tuberculosis* of fragments at each locus, with different numbers of copies, London, UK, 2005–2006*

No. repeats	Length of expected fragments for each locus, bp			
	MIRU 26	ETR-B	VNTR 1982	VNTR 3232†
0	244	121	178	
1	295	174	256	242
2	344	227	334	286
3	393	280	412	330
4	442	333	490	372
5	491	386	568	415
6	540	439	646	458
7	589	492	727	501
8	638	545	802	546
9	687	598	880	587
10	736	651	958	630
11	785		1,038	673
12	834		1,116	716
13	883		1,194	759
14	932			802
15				845
16				888
17				931
18				974
19				1,017
20				1,060

*MIRU, mycobacterial interspersed repetitive unit; ETR, exact tandem repeat; VNTR, variable number tandem repeats. †No isolates had 0 repeats in locus 3232 in our population.

### Assessing Stability and Reproducibility of VNTR Loci

All isolates were grouped into 265 sets of serial isolates (2–6 isolates each) and typed at all 22 loci. Primer sequences for all loci were as described previously (*3*,*9*,*20*,*21*). PCR was set up by using BIOTAQ polymerase for amplifying 12 MIRU and 3 ETR loci and Diamond polymerase for the additional 7 VNTR loci. Capillary electrophoresis was performed by using the parameters described in method 1.

## Results

### Optimization of Hypervariable Loci

We evaluated factors that potentially affect the reproducibility of hypervariable VNTR loci by using various PCR and capillary and manual electrophoresis separation conditions as described in the Materials and Methods. The ability to correctly amplify different VNTR loci depended on the enzyme used (Table 2); all polymerases efficiently amplified MIRU 26 and ETR-B, as indicated by the presence of PCR fragments on agarose gels and capillary electrophoresis peaks. However, locus VNTR 3232 was amplified effectively only with Bioline Diamond (15/16 strains, 93.8%). Although all polymerases except Bioline BIOTAQ were able to amplify DNA at locus VNTR 1982, longer fragments were amplified more efficiently by QIAGEN and Bioline Diamond polymerases. Therefore, Diamond polymerase was selected for the amplification of additional VNTR loci.

**Table 2:** Number of DNA extracts (from n = 16) for which peaks were detected by different conditions for capillary electrophoresis of *Mycobaterium tuberculosis* after amplifying the loci with different polymerases, London, UK, 2005–2006*

Locus	Method†	Bioline polymerases‡			QIAGEN polymerases‡	
		BIOTAQ	Diamond		HotStartTaq	HotStartTaq Plus
MIRU 26	1	16	16		16 (1)	16 (1)
	2	15	16		16 (1)	16 (1)
	3	16	16		16	16
ETR-B	1	16	16		16 (1)	16 (1)
	2	15	16		16 (1)	16 (1)
	3	16	16		16 (2)	16 (2)
VNTR 1982	1	8	13		14	14
	2	9	13		12	14
	3	6	11		12	14
VNTR 3232	1	11	15		13	14
	2	10	15		14	14
	3	11 (3)	15 (7)		13 (6)	13 (4)

*MIRU, mycobacterial interspersed repetitive unit; ETR, exact tandem repeat; VNTR, variable number tandem repeats. †Refer to Table 1. ‡Numbers in parentheses represent number of extracts whose calculated number of repeats were higher and lower than the expected value on the basis of that produced by the standard procedure (method 1).

**Figure 1:**
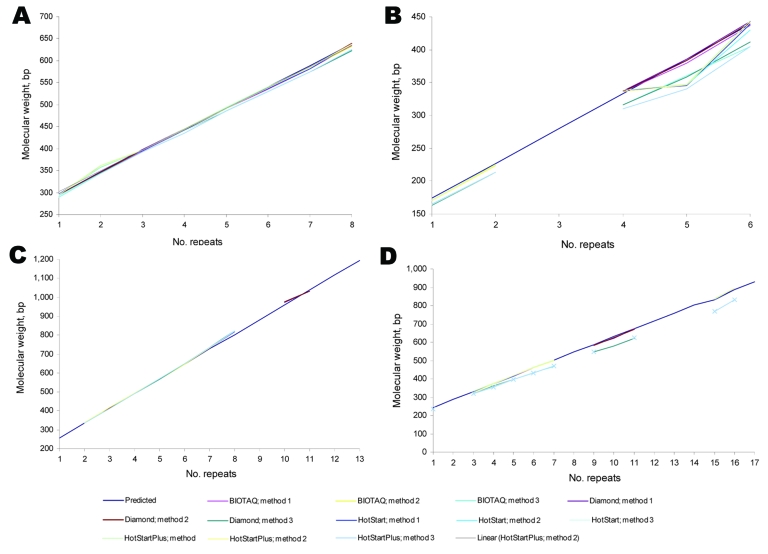
Effect of various enzymes and separation conditions on amplification and detectable molecular weights of PCR fragments for 4 variable number tandem repeat (VNTR) loci. A) Mycobacterial interspersed repetitive unit locus 26; B) locus exact tandem repeat; C) locus 1982; D) locus 3232.

We assessed 3 methods for capillary electrophoresis. For each locus, apparent fragment sizes were plotted against expected fragment sizes for each method (Figure 1).

MIRU 26 fragments sizes were as expected for all allelic variants (except for the variant with 2 repeats) when BIOTAQ and Diamond polymerases were used, but sizes were larger than expected with QIAGEN polymerases. The smaller ETR-B fragments with 1 and 2 repeats all gave expected sizes with methods 1 and 2 but were less than expected with method 3 (where the capillary temperature was decreased). These results did not affect overall interpretation. For the higher number of repeats (4–6 repeats), all polymerases generated fragments that, when analyzed by using method 3, gave apparent sizes lower than expected. In some cases, this result affected the interpretation. The apparent sizes of VNTR 1982 fragments were all similar to the expected values independent of the polymerase used and the method used for capillary electrophoresis.

### Serial Isolates

**Figure 2:**
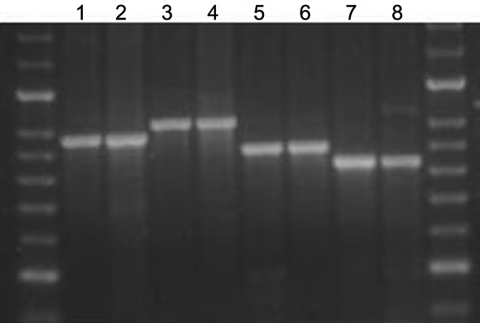
Agarose gel showing the stability of amplified fragments of variable number tandem repeat (VNTR) 3336 from 2 serial isolates isolated from 4 patients. Lane 1, patient A, isolate 1, isolated 2005 Jun 20, 8 copies; lane 2, patient A, isolate 2, isolated 2005 Jul 11, 8 copies; lane 3, patient B, isolate 1, isolated 2005 Jul 8, 9 copies; lane 4, patient B, isolate 2, isolated 2005 Aug 8, 9 copies; lane 5, patient C, isolate 1, isolated 2005 Nov 11, 7 copies; lane 6, patient C, isolate 2, isolated 2005 Nov 15, 7 copies; lane 7, patient D, isolate 1, isolated 2005 May 16, 6 copies; lane 8, patient D, isolate 2, isolated 2005 May 25, 6 copies.

Amplification was performed by using BIOTAQ polymerase for 12 MIRU and 3 ETR loci and Diamond polymerase for 7 VNTR loci with the optimized parameters in method 1. Analysis was blinded. No disagreements occurred in the interpretation of VNTR repeat numbers among isolates in a set. In a proportion of isolates (N = 124), genotyping results were validated by using both capillary electrophoresis and manual electrophoresis for PCR fragment separation, and again, no discrepancies were found between VNTR loci copy numbers in strains isolated from the same patient at different time points (Figure 2).

### Population Genotyping in Metropolitan Setting with 2 Panels of VNTR Loci

A total of 2,261 MTBC isolates circulating in London with known spoligotypes were genotyped by using a defined set of 15 loci (12 MIRU and 3 ETR); all known spoligotyping families were represented in the test population (Technical Appendix). Complete 15-loci profiles were obtained for 2,046 strains (90.5% of all strains). Data for the remaining profiles were incomplete for >1 locus. Overall PCR failure rate was 1.6%, with the highest number of failures (n = 72) at locus ETR-A and the lowest number of failures (n = 4) at locus ETR-C. When PCR failed, DNA was reextracted from original cultures, and genotyping was attempted again. If the second attempt was unsuccessful, the results for the locus were marked as missing.

Genotyping of MTBC isolates by using 15 MIRU-ETR loci yielded 1,036 unique profiles and 235 clusters containing 2–53 isolates (Table 3). Clustered profiles were shared by 1,225 isolates, giving a clustering rate of 54.2%.

**Table 3:** Discriminatory power of VNTR typing used in the study in establishing true minimum cluster size as marker of real transmission rate*

Genotyping method	No. distinct profiles (variety of types)	No. clusters	Size of clusters, no. isolates	Clustering rate, % (n/N)	Recent transmission rate, % ([*n – c*]/*N*)	No. unique isolates
MIRU15 (n = 2261)	1,271	235	2–53	54.2	44.0	1,036
MIRU15 + Spoligotyping	1,619	196	2–48	37.1	29.0	1,423
MIRU15 + VNTR7	1,888	158	2–35	22.2	17.0	1,730

*MIRU, mycobacterial interspersed repetitive unit; VNTR, variable number tandem repeats; n, no. clustered cases; N, total no. of strains; c, no. of clusters.

Subsequently, 1,196 (97.6%) of 1,225 isolates (15 MIRU-ETR clustered isolates) were subjected to secondary typing by using VNTR loci 2163b, 2347, 3232, 2163a, 1982, 3336, and 4052. Resolution improved because strains that had been clustered initially were subdivided into new groups: 1,730 isolates now had unique genotyping patterns, and the remaining 502 isolates were grouped into 158 clusters, giving a new, substantially lower, clustering rate of 22.2% (Table 3).

### Variability and Discriminative Power of VNTR Loci

The discriminative ability of VNTR loci varied markedly among the 22 VNTR loci and among spoligotyping families (Technical Appendix) with locus VNTR 3232 showing the greatest variation (HGDI = 0.909 and 19 allelic variants) and loci MIRU 2 and 20, the least (HGDI = 0.134 and 0.196; number of allelic variants 4 and 3, respectively). Twelve loci each had >10 allelic variants. MIRU 4 showed moderate discriminative power, and MIRU 10, MIRU 16, MIRU 23, MIRU 26, MIRU 40, ETR-A, ETR-C, and VNTR 2163B, 2163A, 1982, 3232, 3336, and 4052 showed high discriminative power with HGDI values varying from 0.524 to 0.909. None of the 22 loci were monomorphic in the current study. With the exception of VNTR 2347, all loci included in the additional VNTR panel displayed higher variability than the primary panel of 15 MIRU-ETR loci used for UK national typing, which indicates their potential for increasing the power of prospective molecular genotyping.

The discriminative power of VNTR loci also varied among spoligotype families. The mean 15 MIRU-ETR HGDI value for the Beijing family was low (0.163), which indicates that this family is relatively homogeneous, even within the diverse London population settings. Notably, mean 15 MIRU-ETR HGDI values for genetic families within the Euro-American lineage (T, Haarlem, S, X, Latin American–Mediterranean) were generally higher (0.307–0.378) than those for Beijing and Central Asian (CAS) (0.235). Within spoligotype families, the additional 7 VNTR increased variability in all cases, except for *M. bovis*. The highest HGDI were seen in the Latin American–Mediterranean family with locus 2163B; in Beijing, Haarlem, and *M. africanum* with VNTR 3232; in East African–Indian with VNTR 2163A; in X with VNTR 1982; in T with VNTR 3336; and in CAS with VNTR 4052. Within the East African–Indian family, the hypervariable loci VNTR 3232 varied little, with 93.7% isolates having a single copy. A small proportion of strains (Table 4) analyzed by using more discriminative loci, including VNTR 3232, 1982, 2163A, and 3336, generated PCR products that were too large for automated analysis but were resolved manually.

**Table 4:** Allelic variants of additional hypervariable VNTR loci that cannot be resolved with the CEQ automated sequencer*†

Locus	Maximum no. repeats suitable for automated analysis	Fragment size, bp	Proportion of strains with allelic variants beyond the automated system resolution, %
3232	15	830	4.1
1982	9	880	11.9
2163A	11	876	10.8
3336	11	875	21.1

*Beckman Coulter, Fullerton, CA, USA. †VNTR, variable number tandem repeats.

## Discussion

Polymorphisms in rapidly evolving repetitive sequences, such as minisatellite VNTR, are a valuable tool for prospective epidemiologic analyses and provide a high degree of discrimination in situations in which few a priori epidemiologic data are available. In this population-based study, we genotyped 2,261 individual MTBC isolates obtained from patients residing in London by using 22 VNTR-MIRU loci.

Conflicting views on the use of hypervariable loci for typing have been reported, even when loci such as VNTR 3232 have been shown to have high discriminatory power (*3*,*5*,*14*). Some studies have demonstrated difficulty in amplification of multiple alleles, absence of PCR amplification products, varying data interpretation, and lack of reproducibility among laboratories (*7*). Similar problems were found with another potentially valuable hypervariable locus, VNTR 1982 (*5*,*7*). Therefore, we believed that by identifying the conditions that provided good, reproducible discrimination, we would be able to define the optimal conditions that would enable molecular epidemiologists to use VNTR 1982 and 3232. We addressed variability and reproducibility for these 2 loci using MIRU 26 and ETR-B as controls that give stable comparable results in both agarose gel and capillary electrophoresis and have been used previously in a multilaboratory comparative study (*7*).

In all cases, identical data were produced for MIRU 26 and ETR-B irrespective of the DNA polymerase used. Amplification of VNTR 1982 and 3232 varied with different DNA polymerases, particularly when expected fragments were long.

The differing performances of polymerases for amplifying different loci can be explained by their varying properties. BIOTAQ polymerase is a basic Taq that can be used for a wide range of templates, whereas Diamond polymerase has been modified by a point mutation at the active site of the enzyme, enabling it to read through regions of secondary structure, microsatellites, and guanine cytosine–rich templates, such as those found in the *M. tuberculosis* genome. The QIAGEN polymerases are chemically modified polymerases with a high specificity similar to that of Diamond polymerase; thus they showed similar capabilities in amplifying VNTR 1982 and 3232. In addition, the buffer used with the QIAGEN polymerases is designed to increase the specificity of primer binding, making these polymerases suitable for dealing with complex genomic DNA.

Conditions that affect the denaturation of PCR products, and therefore their linearity before fragment sizing by electrophoresis, would be expected to influence apparent sizes of PCR fragments and copy number enumeration. We investigated the influence of DNA denaturation time and capillary separation temperature. As expected, we found that lowering the separation rate increased the discrimination of fragments >1,000 bp.

A marked difference was observed when the capillary temperature was decreased (method 3), which was independent of the polymerase used and locus investigated and demonstrated that separation conditions are critical for the correct interpretation of the VNTR typing results. In method 3, apparent fragment sizes were smaller and offset values were markedly larger, to the point that in some cases the calculated copy number was different from that expected.

Taking all the data together, we used BIOTAQ for amplifying MIRU and ETR loci, and Diamond polymerase for amplifying the extra 7 hypervariable VNTR loci, using the separation conditions detailed in method 1. We also demonstrated the reproducibility and stability of the extra 7 VNTR loci by comparing 22 MIRU-VNTR profiles from serial isolates. The resulting profiles of serial isolates from the same patients were identical, indicating that the conditions used for fragment amplification, detection, and analysis were ideal for typing of these loci and that these loci could be used for routine genotyping.

Clustering rates seen by using 15 MIRU-ETR loci far exceeded those previously reported when IS*6110* RFLP was used in a London population study (*22*,*23*). We concluded that 15-MIRU-ETR genotyping was insufficiently discriminative and was producing so-called false clustering. This view was supported by the spoligotyping results in which 38 (16%) of 235 isolates of 15 MIRU-ETR clusters contained isolates that belonged to >2 spoligo families (Table 3).

Applying all 22 loci gave the lowest clustering rate (22.2%) in MTBC strains obtained over 1 year from a single metropolitan setting (London), a rate almost identical to the proportion established in previous studies conducted in London in 1993 and 1995–1997 (*22*,*23*) and similar to previously reported rates in population-based studies in low- to-middle TB incidence settings where RFLP and PCR-based genotyping methods were used (*11*,*24*–*26*). These findings suggest, from the public health viewpoint, that TB transmission in London has remained stable over the past decade. Our study provides strong evidence that PCR-based methods, especially VNTR-MIRU, can replace IS*6110* RFLP typing for prospective analysis and that 12 MIRU (*27*), and 15 MIRU-ETR loci panels alone are insufficiently discriminating for evaluation of TB transmission.

The recently proposed VNTR panel (*3*,*5*,*7*,*11*) provides similar degrees of discrimination (comparable to that achieved by IS*6110* RFLP), although discrimination of individual VNTR loci is not equal for different MTBC genetic families (*13*). Inclusion of highly polymorphic VNTR loci effectively differentiates strains within highly conserved groups and is vital for prospective genotyping. Our study demonstrated that even in settings of low TB incidence and relatively low TB transmission rates, TB families, such as Beijing and CAS, remain more conserved than others, and hypervariable loci (e.g., VNTR 3232, 2163A, 4052) provide much higher discrimination than MIRU and ETR loci either alone or in combination.

Our current results agree with the preliminary results of our earlier studies about the applicability of hypervariable VNTR loci (VNTR 3232, VNTR 3336; VNTR 2163a, and VNTR1982, in particular) and recent reports (*28*–*30*) demonstrating their effectiveness for discrimination among Beijing strains. This agreement suggests that these loci are discriminating and reproducible, especially where Beijing strains are dominant (e.g., China, Russia, Baltic countries) (*28*) and should be included in standardized VNTR panels. They can be used successfully at multiple laboratories with consistent results, provided the conditions for proposed reaction and PCR fragment separation are adhered to and specific DNA polymerases are used.

## Supplementary Material

Technical Appendix Allelic diversity and discriminative values of VNTR loci
