# Supplementary material for: Discriminatory Ability of Hypervariable Variable Number Tandem Repeat Loci in Population-based Analysis of Mycobacterium tuberculosis Strains, London, UK
Source: Emerg Infect Dis. 2009 Oct;15(10):1609–16. doi: 10.3201/eid1510.090463 (PMC2866407; doi:10.3201/eid1510.090463)
Supplement: Technical Appendix — Allelic diversity and discriminative values of VNTR loci [file 09-0463_Techapp-s1.pdf]

Discriminatory Ability of Hypervariable Variable Number Tandem Repeat Loci in Population-based Analysis of *Mycobacterium tuberculosis* Strains, London, UK

Technical Appendix

Allelic diversity and discriminative values of VNTR loci

A. MIRU-ETR loci

Families	HGDI value														
	2	4	10	16	20	23	24	26	27	31	39	40	A	B	C	Mean for 15 loci
EAI (N = 395)	0.01	0.577	0.299	0.363	0.097	0.411	0.115	0.089	0.259	0.637	0.583	0.667	0.788	0.788	0.402	0.406
Beijing (N = 131)	0	0.031	0.296	0.131	0.015	0.018	0.015	0.540	0.354	0.075	0.272	0.377	0.234	0	0.089	0.163
CAS (N = 555)	0.057	0.015	0.701	0.342	0.046	0.053	0.015	0.702	0.044	0.451	0.236	0.49	0.315	0.04	0.014	0.235
T (N = 371)	0.119	0.247	0.693	0.630	0.177	0.393	0.049	0.665	0.197	0.352	0.109	0.751	0.585	0.210	0.497	0.378
Haarlem (N = 207)	0.085	0.131	0.678	0.372	0.325	0.557	0.04	0.413	0.145	0.209	0.076	0.495	0.302	0.315	0.463	0.307
S (N = 20)	0.468	0.485	0.298	0.432	0.426	0.511	0	0.679	0.105	0.433	0.1	0.684	0.526	0.205	0.279	0.375
X (N = 108)	0.262	0.319	0.525	0.073	0.230	0.160	0	0.493	0.091	0.435	0.073	0.731	0.353	0.232	0.704	0.312
LAM (N = 350)	0.173	0.045	0.570	0.600	0.457	0.628	0.011	0.411	0.401	0.211	0.04	0.603	0.567	0.405	0.29	0.361
M. bovis (N = 75)	0	**0.529**	0.054	0.240	0.027	0.053	0.054	0.178	0.207	0.079	0	0.027	0.248	0.335	0.085	0.141
M. afr (N = 46)	0	0.406	0.686	0.605	0.087	0.044	0.390	0.380	0.465	0.641	0	0.489	0.591	0.509	0.555	0.390
All families	0.134	0.524	0.781	0.65	0.196	0.615	0.454	0.797	0.303	0.72	0.559	0.727	0.813	0.551	0.667	-
No of allelic variants	4	11	11	9	3	11	5	11	6	8	4	11	14	9	8	-

B. Additional panel of 7 VNTR loci

Families	HGDI value							
	2163b	2347	3232	2163a	1982	3336	4052	Mean for 7 VNTR loci
EAI (N = 109)	0.804	0.14	0.123	**0.821**	0.796	0.762	0.797	0.606
Beijing (N = 96)	0.737	0.081	**0.830**	0.615	0.399	0.351	0.531	0.506
CAS (N = 332)	0.104	0.042	0.846	0.331	0.635	0.696	**0.855**	0.501
T (N = 208)	0.821	0.055	0.804	0.779	0.651	**0.832**	0.806	0.678
Haarlem (N = 128)	0.826	0.434	**0.935**	0.684	0.760	0.812	0.709	0.737
S (N = 5)	0.4	0	**0.7**	**0.7**	**0.7**	**0.7**	**0.7**	0.557
X (N = 56)	0.616	0.036	0.591	0.478	**0.820**	0.714	0.787	0.577
LAM (N = 252)	**0.880**	0.192	0.8	0.589	0.584	0.826	0.757	0.661
M. bovis (N = 13)	0	0	0	0	0	0.167	0	0.024
M. afr (N = 6)	0.7	0.143	**0.824**	0.681	0.495	0.495	0.813	0.593
All families	0.790	0.382	**0.909**	0.882	0.843	0.887	0.827	-
No. allelic variants	13	6	19	18	14	15	10	-
